# The Biological Role of PI3K Pathway in Lung Cancer

**DOI:** 10.3390/ph5111236

**Published:** 2012-11-20

**Authors:** Evangelos G. Sarris, Muhammad W. Saif, Kostas N. Syrigos

**Affiliations:** 1 Oncology Unit GPP, 3rd Dept of Medicine, Sotiria General Hospital, University of Athens, Athens 11527, Greece; Email: sarris_v@yahoo.gr; 2 Experimental Therapeutics Program, Division of Hematology/Oncology, Tufts Medical Center, Tufts University School of Medicine, Boston, MA 02111, USA; Email: wsaif@tuftsmedicalcenter.org

**Keywords:** lung cancer, PI3K pathway inhibitors, molecular pathways

## Abstract

Lung cancer is the primary cause of cancer-related mortality worldwide and although improvements in treatment have been achieved over the last few years, long-term survival rates for lung cancer patients remain poor. Therefore, there is an imperative need for molecularly targeted agents that will achieve long-term disease control. Numerous downstream molecular pathways, such as EGF/RAS**/**RAF/MEK/ERK and PI3K/AKT/mTOR are identified as having a key role in the pathogenesis of various forms of human cancer, including lung cancer. PI3K/AKT/mTOR signal pathway is an important intracellular signal transduction pathway with a significant role in cell proliferation, growth, survival, vesicle trafficking, glucose transport, and cytoskeletal organization. Aberrations in many primary and secondary messenger molecules of this pathway, including mutations and amplifications, are accounted for tumor cell proliferation, inhibition of apoptosis, angiogenesis, metastasis and resistance to chemotherapy-radiotherapy. In this review article, we investigate thoroughly the biological role of PI3K pathway in lung cancer and its contribution in the development of future therapeutic strategies.

## 1. Introduction

Lung cancer is still the leading cause of cancer-related mortality worldwide. It was estimated that in the United States alone, more than 220,000 new cases and 157,000 deaths occurred due to lung cancer in 2010 [1]. Lung cancer can be classified into two main subtypes: non-small cell lung cancer (NSCLC) and small cell lung cancer (SCLC) with many differences found between these two subtypes concerning the histological type, biological behavior, prevalence, prognosis and response to therapy.

Non-small cell lung cancer accounts for more than 80% of all lung cancer cases. The most common histological types are adenocarcinoma (AC), squamous cell carcinoma (SCC) and large cell lung carcinoma (LCLC). Only a small percentage of patients with NCLC will show early stage disease at the time of presentation and surgery remains the best therapeutic option for these patients [2]. The majority of NSCLC patients are diagnosed at advanced stage with inoperable locally advanced tumors or metastatic disease and the treatment is mainly focused on controlling the disease and sustain life quality, and commonly includes a combination of radio and chemotherapy. However, commonly administered chemotherapy provides no radical treatment for patients with advanced stage disease and has reached a plateau in efficacy with a median survival of 8–10 months [3].

Small cell lung cancer accounts for approximately 13% of all lung cancer cases and is highly associated with tobacco smoking [4,5]. A combination of a platinum agent (cisplatin or carboplatin) and etoposide and in some cases also radiotherapy is the mainstay method in management of SCLC patients [5,6] and although most patients initially respond to chemotherapy, disease recurrence is the most probable outcome and consequently the overall 5-year survival in patients with SCLC is less than 5% [6].

Even though major progress in the understanding of cancer biology and treatment of lung cancer has been achieved over the last few years, the survival rates for both NSCLC and SCLC patients is still disappointing [6,7]. The deregulation of many signaling pathways such as EGF/RAS**/**RAF/MEK/ERK and PI3K/AKT/mTOR is considered to play a critical role in oncogenesis and cancer progression [8]. Therefore, numerous components of these survival pathways may act as potential molecular targets for cancer treatment and the addition of new targeted agents with better tolerability, availability for chronic treatment and better selectivity to conventional chemotherapy has already produced definitive results. Novel therapeutic approaches are urgently needed for this common disease. 

## 2. PI3K Signaling Pathway

**Figure 1:**
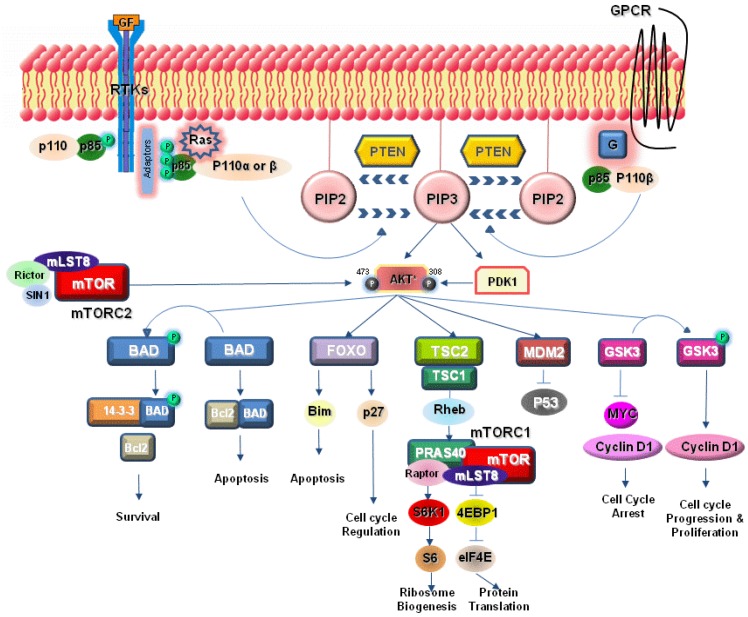
The PI3K/Akt/mTOR signaling pathway.

The phosphoinositide-3-kinase (PI3K) signaling pathway has a critical role in cell growth and survival [9]. Alterations of the PI3K/AKT/mTOR pathway can occur at many levels resulting in PI3K activation and malignant transformation. The PI3Ks are lipid kinases, which can be grouped into three classes based on their structure and function. Class IA PI3K is most closely related to human cancer [10]. Class IA PI3Ks are heterodimers consisting of a regulatory subunit (p85) and a catalytic subunit (p110). Three genes PIK3R1, PIK3R2 and PIK3R3 encode p85α, p85β and p85γ regulatory subunits whereas catalytic isoforms p110α, p110β, and p110δ are the products of three genes PIK3CA, PIK3CB and PIK3CD respectively [10,11,12]. Class IA PI3Ks are usually activated by receptors tyrosine kinase (RTKs) such as EGFR, IGF1-R and HER2/neu [13,14,15,16] and activation often occurs through recruitment of the enzymes to cell membranes via phosphotyrosine binding of the Src-homology 2 (SH2) domains present in the p85 regulatory subunit to the cytoplasmic domains of RTKs. PI3K can be activated also by Ras, which directly binds p110 [17] and the p110β catalytic subunit can be additionally regulated by G-protein coupled receptors [12]. Subsequently, the second messenger phosphatidylinositol-3,4,5-triphosphate (PIP3) is produced through phosphorylation by the activated PI3K of phosphatidyl-inositol-4,5-biphosphate (PIP2). The phosphatase and tensin homolog deleted on chromosome 10 (PTEN) dephosphorylates PIP3 to PIP2, acting thereby as a direct antagonist of PI3K. PIP3 transduces intracellular signaling by directly binding pleckstrin homology (PH) domains of various proteins [16], participating thus in the regulation of cell proliferation and survival, cytoskeletal organization, vesicle trafficking, cell adhesion and motility, angiogenesis and glucose transport [18,19]. Two such PH domain-containing kinases, phosphoinositide-dependent kinase 1 (PDK1) and the serine threonine kinase Akt, are recruited to the membrane via PIP3, where PDK1 activates Akt by phosphorylation at the threonine 308 [20,21]. Mammalian target of rapamycin complex 2 (mTORC2) contributes to the complete activation of Akt via phosphorylation at serine 473. Activated Akt promotes cell growth and survival with various mechanisms. Akt inhibits proapoptotic Bcl-2 family members BAD and BAX [11,16], phosphorylates forkhead box O transcription factors (FoxO), the glycogen synthase kinase 3 (GSK3) and negatively regulates the transcription factor NF-κB, leading to increased expression of antiapoptotic and cell survival signals [22]. The Akt-mediated phosphorylation of TSC2 protein, which combined with TSC1 protein forms a Ras homologue enriched in brain (Rheb) inhibiting complex, allows Rheb to be released and activated. Rheb then stimulates the mammalian target of rapamycin complex 1 (mTORC1), which phosphorylates the p70S6 kinase (S6K1) and the eukaryotic initiation factor 4E binding protein 1 (4EBP1), leading to increased protein synthesis (Figure 1).

## 3. Activation of PI3K Pathway in Lung Cancer

The PI3K pathway is frequently deregulated in lung cancer due to genetic alterations affecting one of its components resulting in increased PI3K signaling [23]. PI3K activation frequently occurs in response to activating mutation and/or amplification of receptors tyrosine kinase (RTK’s), amplification of PI3K, loss or inactivation of PTEN, overexpression of downstream kinase Akt, mutational activation of the PIK3CA gene encoding the p110a catalytic subunit and activation by mutant forms of the Ras oncogene [10,24].

Activating mutations in PIK3CA gene have been described in several tumor types [10,25,26,27,28], and have been usually identified in two key regions in exons 9 (that encodes the helical domain of p110a) and 20 (that encodes the catalytic domain of p110a) [25,27]. However, such somatic mutations are relatively infrequent in lung cancer and appear only in about 5% of NSCLC cell lines [29] and 23% of SCLC cell lines [30]. Transgenic mice, in which mutant p110a was lung-specific induced, carrying mutations in exon 20, developed lung adenocarcinomas [31]. Additionally, there are studies suggesting that the presence of these mutations may be responsible for resistance to agents targeting RTKs [32,33]. Genomic amplification of PIK3CA was also identified in a large number of NSCLC tumors and pre-invasive lesions [34]. Yamamoto *et al*. were not able to identify PIK3CA mutations in SCLC cell lines, but reported PIK3CA copy number gains, associated with increased expression of activated Akt, also identified in 33.1% of squamous cell lung cancer and 6.2% of lung adenocarcinomas [29]. Another study reported PIK3CA gene copy number gain in 76% of SCLC tumors and 54% of SCLC cell lines [35].

Overexpression of the downstream kinase Akt may also result in the PI3K pathway activation. Mutations in AKT1, AKT2, AKT3 genes have been identified in various forms of human cancer but only a limited number of NSCLC tumors harbor mutations in the AKT2 gene responsible for oncogenesis [36], leading to the assumption that the deregulation of the pathway is probably located at a post-transcriptional level. Overactivation of Akt has been reported in NSCLC cell lines, and was closely related to chemo and radioresistance [37], and also in pre-malignant and malignant human bronchial epithelial cells, but not in normal tissue [38]. Activated Akt was also traced in primary NSCLC tumors and was suggested to be a poor prognostic factor for patients with early stage NSCLC [39,40]. Mutant AKT1 gene was reported in 64% of SCLC tumors and 39% of SCLC cell lines [35]. In other studies, high levels of activated Akt were detected in SCLC tumor tissue samples, suggesting the key role of PI3K pathway in disease progression [34,41]. The presence of phosphorylated Akt in SCLC cells that initially developed chemoresistance, supported the hypothesis that activated Akt may be involved in mechanisms responsible for increased chemo and radioresistance [42].

The most common genetic alteration of the PI3K pathway observed in human cancer is deletion or down-regulated expression of the tumor suppressor gene PTEN. PTEN acting as a direct antagonist of PI3K, negatively regulates PI3K pathway. Homozygous or hemizygous deletions of PTEN and missense mutations may result in increased activation of the PI3K pathway and are frequently observed in many cancer types [43,44,45], but are not very frequent in NSCLC [46,47,48,49]. However, partial or complete loss of PTEN protein expression is frequently observed in lung cancer [50,51]. Transcriptional repression and epigenetic silencing of PTEN, commonly through promoter lypermethylation has been described as mechanism of PTEN inactivation in several studies [52,53].

Another downstream regulator of the PI3K pathway, the mammalian target of rapamycin (mTOR), was found to be mutated in more than 30% of 188 lung adenocarcinomas [23] and is also frequently activated in lung cancer cell lines, and especially in these harboring genetic mutations [54,55,56]. There are studies correlating the activation of mTOR with tumor progression and metastatic potential in KRAS-mutated NSCLC models [57]. Anagnostou *et al*. thus reported a better outcome for patients with early stage lung adenocarcinoma that overexpressed mTOR [58].

In many human cancers, RTK’s are often mutated, amplified or overexpressed, resulting in PI3K overactivation. Lung cancers with somatic mutations in epithelial growth factor receptor (EGFR), show EGFR-induced activation of PI3K pathway [31]. When RTK’s-targeted therapies are effective, consequently, PI3K activation is lost and cell death is induced. On the other hand, NSCLC cell lines indicating resistance to tyrosine kinase inhibitor gefitinib, have showed increased levels of PI3K activation [5,59]. Therefore, targeting the PI3K pathway in EGFR-mutant lung cancer showing resistance to TKIs was suggested to be a promising approach [60,61]. Indeed, the class I PI3K-mTOR inhibitor PI-103 was able to induce apoptosis in EGFR-mutant lung cancer cells that initially showed hepatocyte growth factor (HGF)-induced resistance to EGFR-TKIs and in combination with the EGFR inhibitor gefitinib, halt the tumor growth in murine xenograft models [62].

Mutations of the Ras oncogene are also frequently observed in human cancer [63,64]. The GTPase Ras directly bounds p110a subunit resulting in PI3K activation [65]. Mutant p110a subunit, lacking the ability to interact with Ras, inhibited K-Ras-induced lung adenocarcinomas in mice [66]. Moreover, Engelman *et al*. showed that deletion of Pikr1 and Pikr2 was able to halt K-Ras G12D-induced lung oncogenesis [31]. Even though PI3K pathway activation seems to have a key role in K-Ras-induced carcinogenesis, preclinical data suggest that inhibiting PI3K signaling alone may not be entirely effective against K-Ras mutant cancer cell lines or tumors [31,67]. 

## 4. PI3K Pathway Inhibitors in Lung Cancer

Although major progress in the treatment of lung cancer has been achieved over the last few decades, it still remains the cancer type with the highest mortality [1]. Therefore, the need for new therapeutic options with less toxicity, better selectivity and higher effectiveness rises significantly. Small molecule inhibitors (tyrosine kinase inhibitors, TKIs) targeting numerous downstream components of intracellular signal transduction pathways, are prominent therapeutic approaches that have already reached the clinical stage. Targeting PI3K signaling pathway and its downstream mediators is still in early stage, but has already showed promising results and is rapidly processing. In this section, we describe PI3K pathway inhibitors that have reached clinical trials for the treatment of lung cancer, considering four different categories: PI3K inhibitors, dual PI3K- mTOR inhibitors, Akt inhibitors and mTOR inhibitors.

### 4.1. PI3K Inhibitors

The natural product wortmannin and its derivative LY294002 are pan-Class I inhibitors [68,69]. As aforementioned, Akt-mediated activation of the PI3K pathway has been associated with chemo and radioresistance in NSCLC [37]. Even though both compounds were able to increase chemo- and radiosensitivity in NSCLC and SCLC cell lines [37,70,71], they are considered too toxic for human use and have not reached the clinical stage.

PX-866 is also a pan-Class I inhibitor that has the ability to bind PI3K irreversibly [72]. Ihle *et al*. reported the ability of PX-866 to demonstrate antitumor activity *in vivo* against a variety of cancer cell lines [72,73]. Interestingly, cancer cell lines harboring PIK3CA mutations or PTEN loss appeared to be more sensitive to PX-866 [67]. The major toxicity reported was hyperglycemia and decreased glucose tolerance which could be overcome when treated with the antidiabetic agent pioglitazone [74]. In mouse models of oncogenic KRAS-induced lung cancer, PX-866 was able to halt PI3K-induced bronchioalveolar stem cell expansion [75]. The agent is about to enter a Phase I study to determine the maximally tolerated dose (MTD) in combination with docetaxel in patients with solid tumors and a Phase II study to determine the efficacy of PX-866 in combination with docetaxel in patients with NSCLC or Squamous Cell Carcinoma of the Head and Neck (SCCHN) [76] (Table 1).

GDC-0941 is a pan-Class I inhibitor that derives from the pyridofuropyrimidine scaffold and shows high oral availability [77]. It is quickly absorbed and has moderate to long half-life as was shown in a Phase I study [78]. It has already entered clinical trials in patients with solid tumors, with three out of 19 patients showing some level of antitumor activity, and grade 3 headache and pleural effusion to be the dose limiting toxicities (DLTs) observed [79]. The compound will soon enter a Phase I study in combination with paclitaxel and carboplatin (with or without bevacizumab) or pemetrexed, cisplatin, and bevacizumab in patients with advanced NSCLC [80] and in combination with the EGFR inhibitor erlotinib in patients with advanced solid tumors [81]. A Phase II study has already been designed to evaluate the safety and efficacy of GDC-0941 in combination with carboplatin-paclitaxel or carboplatin-paclitaxel and bevacizumab in patients with previously untreated advanced or recurrent NSCLC [82]. (Table 1)

XL-147 (SAR245408) is a pan-Class I inhibitor with long half-life and prolonged absorption. In a Phase I study in patients with solid tumors treated orally once a day with XL-147, thirteen patients remained on trial for more than 16 weeks and one patient with NSCLC showed partial response (PR) by RECIST criteria. The compound demonstrated reduction in PI3K and MEK/ERK pathway signaling and prolonged stable disease. Grade 3 rash and grade 4 arterial thrombosis were the serious adverse events (SAEs) observed in the trial, with skin rash to be the most common drug-related toxicity [83]. Currently, the entity is in a Phase I study in combination with paclitaxel and carboplatin in adults with solid tumors including NSCLC patients [84] (Table 1) and has already completed a Phase I study in combination with the EGFR inhibitor erlotinib in patients with solid tumors in which 8 patients with NSCLC were enrolled. The combination of XL-147 and erlotinib was well tolerated (rash, nausea, diarrhea, fatigue and vomiting were the most frequently observed toxicities) in dose levels up to 400 mg XL147 and 150 mg erlotinib daily and showed parallel inhibition of PI3K and EGFR signaling [85].

NVP-BKM120 is a highly specific, orally available pan-Class I PI3K inhibitor that has already completed a Phase I dose-escalation study in patients with advanced malignancies. In this study, thirty-five patients were enrolled and orally treated with NVP-BKM120 daily. The MTD was set at 100 mg/day. The most frequent drug-related adverse events were rash, hyperglycemia, diarrhea, anorexia, and mood alteration. Seven of 35 patients remained in the study for over 8 months and the entity was able to present preliminary antiproliferative activity [86]. The combination of NVP-BKM120 with the mTOR inhibitor rapamycin resulted in synergistic growth inhibition in NSCLC cell lines. Moreover, NVP-BKM120 when combined with the mTOR inhibitor RAD001 (Everolimus), managed to inhibit the growth of lung cancer cells *in vitro* and also in murine lung cancer xenograft models [87]. Currently, NVP-BKM120 is in a Phase I study with the EGFR inhibitor gefitinib in patients with advanced NSCLC particularly enriched with patients harboring alterations of the PI3K pathway and overexpress EGFR [88] and also in a Phase I/II trial with the EGFR inhibitor erlotinib in patients previously sensitive to erlotinib [89]. NVP-BKM120 is undergoing a Phase II study with docetaxel or docetaxel and pemetrexed in patients with metastatic NSCLC [90] and a Phase I study in combination with the mTOR inhibitor everolimus in patients with advanced solid tumors [91]. Lastly, a Phase II study of NVP-BKM120 is currently being conducted in patients with PIK3CA activating mutations [92] (Table 1).

### 4.2. Dual PI3K-mTOR Inhibitors

Chemical compounds that have the ability to inhibit both mTOR and the p110 catalytic subunits are termed dual PI3K- mTOR inhibitors. These inhibitors have the possible advantage of multi-blocking the PI3K pathway, even though it is still unclear if they can effectively inhibit all p110 isoforms and mTORC1- mTORC2 in doses tolerable for clinical use.

NVP-BEZ235 is an imidazo-quinoline derivative, orally available, that belongs to the family of dual PI3K-mTOR inhibitors [93,94]. It was the first entity of this class to enter Phase I studies in patients with advanced solid tumors (many patients with breast cancer were enrolled) in which NVP-BEZ235 showed efficacy and anti-tumor activity [95]. NVP-BEZ235 was able to achieve a decrease in cell proliferation and G1 cell cycle arrest in a variety of cancer cell lines and halt further tumor growth in xenograft models of these cancer types [31,93,96,97]. Compared to mTORC1 inhibitor rapamycin, NVP-BEZ235 was more efficient in blocking tumor cell growth [98]. Moreover, NVP-BEZ235 was able to show anti-tumor efficacy *in vitro* and *in vivo* and also increase radiosensitivity in KRAS-mutant NSCLC cell lines [96]. Another study with genetically engineered mice demonstrated that even though the compound, as single-agent, failed to inhibit murine KRAS-mutant lung tumors, when combined with a MEK inhibitor (ARRY-142886) resulted in tumor shrinkage [31]. In the same study, NVP-BEZ235 was highly effective at shrinking a murine lung adenocarcinoma with a somatic mutation in the p110α kinase domain (H1047R) [31]. These results led to the assumption that lung cancer tumors harboring PIK3CA mutations could benefit from the inhibition of PI3K signaling and the combination of both PI3K and MEK inhibitors might show efficacy in KRAS-mutant lung cancers [31]. Sos ML et al. using a panel of NSCLC cell lines, confirmed that tumors with activating mutations in RTKs present high dependence on PI3K signaling and mutations in the RAS/RAF pathway is strongly correlated with MAPK signaling [99]. In another study, EGFR-mutant NSCLC models did not respond to single-agent NVP-BEZ235, but when combined with a MEK inhibitor (AZD6244), tumor regression could be observed suggesting the key role of simultaneous PI3K and MEK inhibition in lung cancers with EGFR mutations [100].

XL-765 (SAR245409) is another dual PI3K-mTOR inhibitor that recently completed a Phase I dose-escalation study in patients with advanced solid tumors. The compound was orally administered and well tolerated with elevated hepatic enzymes, with nausea and diarrhea being the most frequent drug-related adverse events (AEs). No partial responses were observed, but five of 36 patients presented stable disease, one of them with NSCLC. Evidence of 60% to 90% pathway inhibition was found in hair and skin [101]. The combination of XL-765 with the EGFR inhibitor erlotinib was tested in a Phase I study in 21 patients with advanced solid tumors, including 14 patients with NSCLC. The combination was well tolerated in doses up to 50 mg XL-765 and 100 mg erlotinib with skin and subcutaneous tissue disorders (including rash) and diarrhea to be the most commonly observed treatment-related adverse events (AEs) and showed satisfactory dual PI3K and EGFR signaling inhibition [102]. 

Another dual PI3K-mTOR inhibitor PI-103, a pyridinylfuranopyrimidine compound, was able to induce apoptosis in NSCLC cell lines with resistance to EGFR inhibitor gefitinib [103]. Cell lines harboring activating mutations of the PIK3CA gene were more sensitive than wild-type PIK3CA cancer cell lines [103]. Moreover, PI-103 increased sensitivity to radiation in tumor cells *in vitro* [104] and caused vascular normalization in murine xenograft models *in vivo* [105]. The entity is still under clinical evaluation.

### 4.3. Akt Inhibitors

Akt inhibitors are chemical agents based on staurosporine and derivatives that have the ability to block the serine/threonine kinase Akt, crucial component of the PI3K pathway [106,107]. However, preclinical data suggest that parallel inhibition of both Akt1 and Akt2 could result in peripheral insulin resistance and drug-induced, dose-dependent hyperglycemia and hyperinsulinemia [108]. In several studies using mouse models, Akt inhibitors have been implicated for causing hyperglycemia [109,110], supporting the hypothesis of having a key role in insulin signaling and glucose homeostasis. Therefore, concerns have been raised for possible limitation in the therapeutic applications of Akt inhibitors due to metabolic toxicities.

MK-2206 is an orally administered pan-AKT kinase inhibitor presenting high selectivity for Akt. Preclinical data demonstrated the ability of MK-2206 to inhibit cancer cell proliferation when combined with cytotoxic agents (such as docetaxel, doxorubicin, gemcitabine, 5-FU and carboplatin) or targeted therapeutic agents (such as erlotinib or lapatinib) [111]. In a Phase I study, thirty-three patients with solid tumors were treated with MK-2206, establishing the MTD at 60 mg. One patient with pancreatic adenocarcinoma presented 60% reduction in cancer antigen 19-9 levels and 23% tumor shrinkage. Skin rash, nausea, pruritus, hyperglycemia and diarrhea were the most commonly observed adverse events [112]. Currently, a dose-escalation Phase I study of MK-2206 combined with the EGFR-TKI gefitinib is being conducted in patients with NSCLC, particularly enriched with patients harboring EGFR mutations [113]. Also, a Phase II study of MK-2206 and the EGFR inhibitor erlotinib is currently recruiting patients with NSCLC who have progressed after previous response to erlotinib, in order to assess the safety of the drug combination [114] (Table 1). Other Akt inhibitors such as A-443654 and GSK690693 are currently under clinical evaluation [109,115].

### 4.4. mTOR Inhibitors

Compounds targeting the mTOR pathway can be grouped into two main subtypes: the allosteric mTOR inhibitors (like rapamycin and its derivatives) and the ATP-competitive mTOR inhibitors.

Rapamycin (sirolimus, Rapamune^®^), a macrolide isolated from *Streptomyces hygroscopicus*, is an allosteric inhibitor of the mTORC1 complex (but not mTORC2) with antifungal, immunosuppressive and antiproliferative activity [116]. Even though the anti-tumor efficacy of rapamycin is well documented both *in vitro* and *in vivo*, it is still not entirely understood. By reducing the levels of cyclins (especially cyclin D) and increase the levels of cyclin-dependent kinase inhibitors p21^cip1^ and p27^kip1^, rapamycin blocks G1 cell cycle progression [117,118,119,120]. The compound presents also anti-angiogenic properties by inhibiting endothelial cell proliferation, reducing the levels of produced vascular endothelial growth factor (VEGF) and reducing the response of endothelial cells to VEGF via mTOR inhibition [121,122,123]. There are preclinical data suggesting compounds’ ability to block the growth of human NSCLC cells and inhibit the growth of a Ras-induced NSCLC tumor and alveolar epithelial neoplasia [124,125,126]. The combination of rapamycin and docetaxel was found to synergistically inhibit the growth of lung cancer cells [127] and also the addition of rapamycin to PI3K inhibitors LY294002 and NVP-BKM120 resulted in synergistic act against NSCLC specimens [128], suggesting the possible efficacy of using rapamycin in combination with chemotherapy or other targeted agents in the treatment of lung cancer. However, the unfavorable pharmacological properties of rapamycin have promoted the discovery and development of rapamycin analogues suitable for clinical use, such as everolimus (RAD001), temsirolimus (CCI-779) and deforolimus (AP23573). 

CCI-779 (temsirolimus) is an orally available rapamycin analogue with significant antiproliferative activity against a variety of human cancer types including SCLC [129]. Ohara *et al*. recently reported the ability of temsirolimus to inhibit tumor cell proliferation in NSCLC cell lines in a dose-dependent manner [130]. In a Phase I dose-escalation study, CCI-779 was i.v. administered in 63 patients with solid tumors. One patient with NSCLC had a confirmed partial response (PR) maintained for 12.7 months, three out of 63 patients had unconfirmed PRs and in two patients stable disease was observed for over 24 weeks. The most frequent drug-related toxicities were fatigue, mucositis and nausea and the maximally tolerated dose was 15 mg/m^2 ^for heavily pretreated patients and 19 mg/m^2^ for patients with minimal prior treatment [131]. In another Phase I trial, patients with advanced solid tumors were orally treated with CCI-779 and the MTD was set at 75 mg. Elevated liver enzymes and rash were the dose-limiting toxicities observed in the study, and mucositis, rash and asthenia the most commonly identified drug-related adverse events [132]. Temsirolimus was also administered in 87 patients with extensive-stage SCLC in remission after induction chemotherapy. The overall median progression-free survival (PFS) time was 2.2 months and the median overall survival (OS) time was 8 months. Only one patient experienced a PR and six patients achieved disease stabilization, thus temsirolimus failed to prolong PFS in stable or responding patients with extensive-stage SCLC after induction chemotherapy [133]. Fifty-five patients with advanced NSCLC received CCI-779 i.v. at a dose of 25 mg/week in a Phase II study. Four patients had confirmed PR and SD maintained for 8 weeks or more was observed in fourteen patients. Dyspnea, fatigue, hyperglycemia, hypoxia, nausea and rash were the most frequent drug-related adverse events reported [134]. In a Phase I dose-escalation study, CCI-779 was tested in combination with the EGFR inhibitor EKB-569 in 48 patients with advanced solid tumors. The MTD was established at 30 mg on days 1–3 and 15–17 in a 28-day cycle for temsirolimus and 35 mg daily for EKB-569. The most common grade 3/4 toxicities observed were diarrhea, dehydration, and nausea-vomiting. Four out of 48 patients had a partial response and 15 patients showed stable disease [135]. Currently, CCI-779 is undergoing numerous clinical studies either as a single agent or in combination with cytotoxic agents (such as pemetrexed, vinorelbine and docetaxel) in patients with lung cancer [136,137,138] (Table 1).

RAD001 (everolimus) is an orally available rapamycin derivative that has already been approved in Europe as an immunosuppressive agent to prevent rejection in adult cardiac and renal transplant patients [139,140]. RAD001 showed significant antitumor activity in cancer cell lines and xenograft models of various cancer types including lung cancer [141,142,143,144,145]. Everolimus has the ability to bind with high affinity to FKB12, the intracellular receptor for mTOR, form a complex that interacts with mTOR and consequently halt downstream signaling [143].

RAD001 was evaluated in a Phase II nonrandomized study comparing NSCLC patients with two or fewer prior lines of chemotherapy, one platinum-based (arm 1) to those who failed second line chemotherapy in combination with an EGFR inhibitor (arm 2). Eighty-five patients (42 in arm 1 and 43 in arm 2) were enrolled and treated with Everolimus at a dose of 10 mg/day until progression disease or unacceptable toxicity. The overall response rate (ORR) was 4.7% and the overall disease control rate was 47.1%. The median progression-free survival (PFS) was 2.6 months in arm 1 and 2.7 months in arm 2. The most commonly observed toxicities were fatigue, dyspnea, stomatitis, anorexia, anemia and thrombocytopenia [146].

In a Phase I trial, RAD001 was orally administered in combination with the EGFR inhibitor gefitinib in patients with advanced NSCLC. Ten patients were enrolled in this study and the maximum tolerated dose of everolimus was set at 5 mg/day when coadministered with 250 mg gefitinib daily. At this dosage, the combination of RAD001 and gefitinib had only mild to moderate toxicities. When everolimus was administered at a higher dose, two dose-limiting toxicities were observed (grade 5 hypotension and grade 3 stomatitis). Among the eight evaluated patients, only two confirmed partial responses (PRs) were identified [147]. The combination of everolimus and gefitinib was also evaluated in a Phase II study in which 62 NSCLC patients participated. The patients were stratified into two cohorts based on whether they had been previously treated with cisplatin or carboplatin and docetaxel or pemetrexed (arm 2) or if they had received no prior treatment (arm 1). Only in 8 out of 62 patients, PRs were identified, thus the overall response rate was 13%. The median time to progression was 4 months and the median overall survival was 12 months, 27 months in arm 1 and 11 months in arm 2. Even though the combination was well tolerated with only mild to moderate toxicities, the partial response rate observed did not meet the predefined response to justify further investigation [148].

Campone *et al*. conducted a Phase I study of everolimus and paclitaxel weekly administered in patients with solid tumors. Sixteen patients were enrolled, eleven of whom achieved disease stabilization. The main DLT observed in this study was myelosuppression [149].

Twenty-four patients with advanced NSCLC and progression after platinum-based chemotherapy were enrolled in a Phase I study of RAD001 in combination with docetaxel. The DLTs were fever with grade 3/4 neutropenia, grade 3 fatigue and grade 3 mucositis. Among 21 patients evaluated, one patient with lung adenocarcinoma had a PR and 10 patients achieved disease stabilization. The recommended Phase II doses for the combination are 60 mg/m^2^ for docetaxel and 5 mg daily for everolimus [150].

The combination of everolimus and pemetrexed was also tested in a Phase I dose-escalation study in patients with NSCLC who progressed after one prior treatment. Forty-three patients were enrolled and in 5 of them, PR was identified. Everolimus 5 mg daily or 50 mg weekly in combination with the standard regimen of pemetrexed, was well tolerated and the most common grade 3/4 adverse events observed were neutropenia, dyspnea and thrombocytopenia [151].

Everolimus and the EGFR inhibitor erlotinib were administered in patients with advanced NSCLC previously treated with chemotherapy in a Phase I trial. Sixty-one patients were enrolled in the study and the drug-combination was well tolerated with mucositis, rash, diarrhea, vomiting and neutropenia to be the DLTs observed. One patient had complete response (CR), three patients had PR and 17 patients presented stable disease [152]. The combination was further tested in a Phase II study in which 133 patients participated. Even though the combination arm of everolimus plus erlotinib had 11% better disease control rate (DCR) at 3 months than the single-agent erlotinib arm, it did not meet the prespecified threshold for a Phase III study [153].

In a Phase I clinical study, RAD001 will be tested in patients with operable NSCLC [154]. In another Phase I study, the combination of everolimus and carboplatin-paclitaxel with or without bevacizumab is being evaluated in patients with NSCLC [155] (Table 1).

In SCLC cell lines RAD001 were able to inhibit cell growth *in vitro* and also in xenograft models of SCLC [156,157]. Moreover, SCLC cell lines exhibiting overactivation of the Akt/mTOR signaling were shown to be more sensitive to RAD001 treatment, suggesting the possible key role of inhibiting mTOR in patients with SCLC [156]. In a Phase II study, 10 mg everolimus were administered daily to 40 previously treated patients with SCLC after progression. In 35 evaluated patients, one had PR, and in eight patients disease stabilization was identified. The disease control rate at 6 weeks was 26%, the median time to progression was 1.3 months and the median survival was 6.7 months. Thrombocytopenia, neutropenia, infection, pneumonitis, fatigue, elevated transaminases, diarrhea and acute renal failure were the grade 3 adverse events observed. Even though the entity was well tolerated in this study, it had only moderate efficacy in pre-treated patients with relapsed SCLC [158].

Preclinical data suggest that everolimus and the EGFR inhibitor erlotinib have synergistic effect in atypical bronchial carcinoids (AC) and large cell neuroendocrine lung carcinomas (LCNEC), indicating the clinical importance of EGFR and mTOR as therapeutic targets in bronchial neuroendocrine tumors [159]. A Phase II study of everolimus with paclitaxel and carboplatin as first line treatment in patients with advanced large cell lung cancer with neuroendocrine differentiation is being conducted [160] (Table 1).

Currently, everolimus is in a Phase I study in combination with paclitaxel in patients with relapsed or refractory SCLC [161] and recently a dose-escalation Phase I study of everolimus in combination with carboplatin and etoposide in patients with SCLC or other advanced malignancies was terminated due to increased number of toxicities observed in the trial [162]. The combination of everolimus and cisplatin-etoposide is being evaluated in a Phase I trial in non-previously-treated patients with extensive-stage SCLC [163] (Table 1).

Drug-related pulmonary toxicity has been described for mTOR inhibitors in several studies and has been reported to be as high as 25% to 36% with typical radiographic findings, including lung consolidation and nonspecific areas of ground glass attenuation [164,165]. Patients with mTOR pneumonitis can be asymptomatic or have only mild symptoms, thus careful monitoring is required and treatment with mTOR inhibitors can often be continued. 

AP23573 (ridaforolimus) is a rapamycin analogue and a small molecule mTOR inhibitor. The compound has shown significant antiproliferative activity in various human cancer cell lines and murine xenografts, as a single agent or in combination with cytotoxic or targeted agents [166,167]. In a Phase I study, ridaforolimus was administered to 13 Japanese patients with advanced solid tumors, and was well tolerated up to a dose of 40 mg. The most common adverse events identified were stomatitis, hypertriglyceridemia and proteinuria. In this study, one patient with NSCLC experienced PR [168]. In another dose-escalation Phase I study, thirty-two patients with advanced tumors received AP23573 intravenously daily for 5 days every 2 weeks in a 28-day cycle. The entity was well tolerated and the maximum-tolerated dose (MTD) was 18.75 mg/d. One patient with NSCLC had PR [169].

Up to date, a Phase II study of AP23573 in NSCLC patients with KRAS-mutations is ongoing [170] and a Phase I study of AP23573 combined with cetuximab in patients with NSCLC, head and neck cancer and colon cancer [171] (Table 1).

ATP competitive mTOR inhibitors have the ability of blocking both mTORC1 and mTORC2 complex producing a more significant antitumor activity compared to rapamycin derivatives [172].

AZD8055 is an ATP competitive mTOR inhibitor that has already completed a Phase I study in patients with solid tumors and lymphomas. Forty-nine patients were treated with AZD8055 and the most frequently observed drug-related adverse events were increased transaminases and fatigue. The maximum tolerated dose was set at 90 mg. Even though seven patients had stable disease maintained for over 4 months, no responses by RECIST criteria were identified [173]. Preclinical data suggest that AZD8055 and the MEK inhibitor selumetinib have synergistic antitumor efficacy in murine xenograft models of human lung adenocarcinomas [174]. Other ATP competitive mTOR inhibitors such as KU-0063794, WYE-354, WYE-132, OXA-01 are currently under clinical research in patients with solid tumors including lung cancer patients.

**Table 1:** Ongoing trials with PI3K pathway inhibitors in the treatment of lung cancer.

Title	Phase	Protocol ID	Cancer type	Compounds	Mechanism
Study of PX-866 and Docetaxel in Solid Tumors [76]	Phase I + Phase II	NCT01204099	Solid tumors(NSCLC)	PX-866 + Docetaxel	PI3K inhibitor
A Study of the Safety and Pharmacology Of PI3-Kinase Inhibitor GDC-0941 In Combination With Either Paclitaxel And Carboplatin (With or Without Bevacizumab) or Pemetrexed, Cisplatin, And Bevacizumab in Patients With Advanced Non Small Cell Lung Cancer [80]	Phase I	NCT00974584	NSCLC	GDC-0941 + Paclitaxel + Carboplatin(with or without Bevacizumab) or Pemetrexed + Cisplatin + Bevacizumab	PI3K inhibitor
A Study of the Safety and Pharmacology of GDC-0941 in Combination With Erlotinib in Patients With Advanced Solid Tumors [81]	Phase I	NCT00975182	Solid tumors	GDC-0941 + Erlotinib	PI3K inhibitor
Study Evaluating the Safety and Efficacy Of Carboplatin/Paclitaxel And Carboplatin/Paclitaxel/Bevacizumab With and Without GDC-0941 in Patients With Previously Untreated Advanced Or Recurrent Non-small Cell Lung [82]	Phase II	NCT01493843	NSCLC	Carboplatin + Paclitaxel or Carboplatin + Paclitaxel + Bevacizumab with and without GDC-0941	PI3K inhibitor
Safety Study of XL147 (SAR245408), in Combination With Paclitaxel and Carboplatin in Adults With Solid Tumors [84]	Phase I	NCT00756847	Solid tumors	XL-147 + Paclitaxel + Carboplatin	PI3K inhibitor
A Trial of Gefitinib in Combination With BKM120 in Patients With Advanced Non-Small Cell Lung Cancer, With Enrichment for Patients Whose Tumors Harbour Molecular Alterations of PI3K Pathway and Known to Overexpress EGFR [88]	Phase I	NCT01570296	NSCLC	BKM120 + Gefitinib	PI3K inhibitor
Trial of Erlotinib and BKM120 in Patients With Advanced Non Small Cell Lung Cancer Previously Sensitive to Erlotinib [89]	Phase I+Phase II	NCT01487265	NSCLC	BKM120 + Erlotinib	PI3K inhibitor
Safety and Efficacy of BKM120 in Patients With Metastatic Non-small Cell Lung Cancer [90]	Phase II	NCT01297491	NSCLC	BKM120 + Docetaxel orDocetaxel + Pemetrexed	PI3K inhibitor
A Phase I Study of BKM120 and Everolimus in Advanced Solid Malignancies [91]	Phase I	NCT01470209	Solid tumors	BKM120 + Everolimus	PI3K inhibitor
BKM120 in Cancers With PIK3CA Activating Mutations [92]	Phase II	NCT01501604	Solid tumors with PIK3CA mutations	BKM120	PI3K inhibitor
Dose Defining Study For MK-2206 Combined With Gefitinib In Non Small Cell Lung Cancer (NSCLC) [113]	Phase I	NCT01147211	NSCLC	MK-2206 + Gefitinib	Akt inhibitor
MK2206 and Erlotinib Hydrochloride in Treating Patients With Advanced Non-Small Cell Lung Cancer Who Have Progressed After Previous Response to Erlotinib Hydrochloride Therapy [114]	Phase II	NCT01294306	NSCLC	MK-2206 + Erlotinib	Akt inhibitor
Temsirolimus and Pemetrexed for Recurrent or Refractory Non-Small Cell Lung Cancer [136]	Phase I + Phase II	NCT00921310	NSCLC	Temsirolimus + Pemetrexed	mTOR inhibitor
Temsirolimus and Vinorelbine Ditartrate in Treating Patients With Unresectable or Metastatic Solid Tumors [137]	Phase I	NCT01155258	Solid tumors	Temsirolimu + Vinorelbine	mTOR inhibitor
Phase I Study of Docetaxel and Temsirolimus in Resistant Solid Malignancies [138]	Phase I	NCT00703625	Solid tumors	Temsirolimus + Docetaxel	mTOR inhibitor
Phase 1b Trial of RAD001 in Patients With Operable Non-Small Cell Lung Cancer (NSCLC) [154]	Phase I	NCT00401778	NSCLC	Everolimus	mTOR inhibitor
Combination of RAD001 With Carboplatin, Paclitaxel and Bevacizumab in Non-small-cell Lung Cancer (NSCLC) Patients [155]	Phase I	NCT00457119	NSCLC	Everolimus + Carboplatin + Paclitaxel + Bevacizumab	mTOR inhibitor
RAD001 With Paclitaxel and Carboplatin in First Line Treatment of Patients With Advanced Large Cell Lung Cancer With Neuroendocrine Differentiation [160]	Phase II	NCT01317615	LCLC	Everolimus + Paclitaxel + Carboplatin	mTOR inhibitor
Combination Anticancer Therapy of Paclitaxel and Everolimus for Relapsed or Refractory Small Cell Lung Cancer [161]	Phase I	NCT01079481	SCLC	Everolimus + Paclitaxel	mTOR inhibitor
Everolimus, Carboplatin, and Etoposide in Treating Patients With Small Cell Lung Cancer or Other Advanced Solid Tumors [162]	Phase I	NCT00807755	SCLC(Solid tumors)	Everolimus + Carboplatin + Etoposide	mTOR inhibitor
Safety of RAD001 in Combination With Cisplatin and Etoposide in Lung Cancer Patients [163]	Phase I	NCT00466466	SCLC	Everolimus + Cisplatin + Etoposide	mTOR inhibitor
A Study of Ridaforolimus in Non-Small Cell Lung Cancer (NSCLC) Patients With Kirsten Rat Sarcoma Viral Oncogene Homolog (KRAS) Mutations (MK-8669-021 AM1) [170]	Phase II	NCT00818675	NSCLC	Ridaforolimus	mTOR inhibitor
Ridaforolimus With Cetuximab for Patients With Advanced Head and Neck Cancer, Non-Small Cell Lung Cancer and Colon Cancer [171]	Phase I	NCT01212627	Solid tumors	Ridaforolim + Cetuximab	mTOR inhibitor

## 5. Conclusions

Even though major progress has been made in the treatment of patients with lung cancer, the survival rates remain poor. The importance of intracellular signal transduction pathways such as PI3K/AKT/mTOR pathway in cell growth, survival and proliferation has been justified over the last few years. The overactivation of such pathways has been identified in many cancer types including lung cancer and is strongly correlated with tumor development and progression, metastasis, chemo and radioresistance. Many downstream regulators of PI3K pathway have become targets for cancer treatment with encouraging results up to date. Indeed, numerous targeted agents directly against the PI3K pathway have already reached the clinical stage either as single agents or in combination with conventional chemotherapy or other targeted therapies, presenting a much better toxicity profile compared to conventional chemotherapy. Many frequently observed side effects, such as peripheral insulin resistance deriving from the use of Akt-inhibitors, are expected and can be justified by the mechanism of action of these agents. Moreover, small molecule agents with the ability to inhibit various signaling pathways in parallel seem to be more effective compared to single-target agents. More clinical trials along with the identification of biomarkers, able to characterize the “PI3K activated” tumors and predict clinical benefit from the use of PI3K pathway inhibitors, are required in order to produce more definite results for this fatal disease.
